# Genome sequencing and application of Taiwanese macaque *Macaca cyclopis*

**DOI:** 10.1038/s41598-023-38402-4

**Published:** 2023-07-17

**Authors:** Kuo-Ping Chiu, Lutimba Stuart, Hong Sain Ooi, John Yu, David Glenn Smith, Kurtis Jai-Chyi Pei

**Affiliations:** 1 Genomics Research Center, Academia Sinica, Taipei, Taiwan; 2 Top Science Biotechnologies, Inc., 4F, 50-2 Dingping Rd., Sec. 1, Shiding District, New Taipei City, 223002 Taiwan; 3 Institute of Stem Cell and Translational Cancer Research, Chang Gung Memorial Hospital at Linkou, No.5, Fu-Shin St., Kuei Shang, Taoyuan, 333 Taiwan; 4 Department of Anthropology, University of California Davis, Davis, CA USA; 5 Institute of Wildlife Conservation, College of Veterinary Medicine, National Pingtung University of Science and Technology, Pingtung, Taiwan

**Keywords:** Computational biology and bioinformatics, Genetics

## Abstract

Formosan macaque (*Macaca cyclopis*) is the only non-human primate in Taiwan Island. We performed de novo hybrid assembly for *M. cyclopis* using Illumina paired-end short reads, mate-pair reads and Nanopore long reads and obtained 5065 contigs with a N50 of 2.66 megabases. *M. cyclopis* contigs >  = 10 kb were assigned to chromosomes using Indian rhesus macaque (*Macaca mulatta mulatta*) genome assembly Mmul_10 as reference, resulting in a draft of *M. cyclopis* genome of 2,846,042,475 bases, distributed in 21 chromosomes. The draft genome contains 23,462 transcriptional origins (genes), capable of expressing 716,231 exons in 59,484 transcripts. Genome-based phylogenetic study using the assembled *M. cyclopis* genome together with genomes of four other macaque species, human, orangutan and chimpanzee showed similar result as previously reported. However, the *M. cyclopis* species was found to diverge from Chinese *M. mulatta lasiota* about 1.8 million years ago. Fossil gene analysis detected the presence of *gap* and *pol* endogenous viral elements of simian retrovirus in all macaques tested, including *M. fascicularis*, *M. m. mulatta* and *M. cyclopis*. However, *M. cyclopis* showed ~ 2 times less in number and more uniform in chromosomal locations. The constrain in foreign genome disturbance, presumably due to geographical isolation, should be able to simplify genomics-related investigations, making *M. cyclopis* an ideal primate species for medical research.

## Introduction

The genus *Macaca* constitutes the Cercopithecidae subfamily of Old World Monkeys formed by ~ 20 macaque species spreading across Asia and North Africa^1^. Based on male external genitalia, Fooden classified these macaques into four groups: *fascicularis, silenus-sylvanus*, *sinica* and *arctoides*^2^. As the largest group of macaques, the *fascicularis* group spreads across a wide geographic area^3,4^, suggesting a vast genetic and phenotypical diversity. This group comprises *M. cyclopis* (Taiwanese macaque, Formosan macaque, or Formosan rock macaque), *M. fascicularis* (cynomolgus/crab-eating macaque)*, M. fuscata* (Japanese macaque) and *M. mulatta* which includes Indian rhesus monkey (*Macaca mulatta mulatta*) and Chinese rhesus monkey (*M. mulatta lasiota*). The *silenus-sylvanus* group comprises *M. silenus, M. sylvanus, M. nemestrina* and Sulawesi species, while the *sinica* group includes *M. sinica, M. assamensis, M. radiate* and *M. thibetana* and the *arctoides* group includes *M. arctoides*. Guided by the Alu element distribution pattern, Li and colleagues split *sylvanus* from *silenus* and assigned *arctoides* to the *sinica* group^5^. Studies based on territorial distribution pattern suggested that the *silenus* group diverged before the *fascicularis* group^2,5^.

Macaque species possess a strong potential for the study of primate evolution, human physiology and diseases (e.g., cancers and viral infections) due to the fact that they are very close to humans in both genetic makeup and evolution. During mid-twentieth century, millions of *M. m. mulatta* were exported from India to many countries, especially USA, to be used for medical and biological studies. The exporting activity resulted in a dramatic reduction of the population and was thus prohibited in 1978^6^, causing a shortage of Indian rhesus monkey for medical and biological studies. Endemic to Taiwan, *M. cyclopis* is the only non-human primate in the island. Due to legislative protection act for the past few decades, the *M. cyclopis* population has increased. There is a need to learn more about this primate species so to prepare for its potential usage in the future.

Genome sequencing aims to provide a genetic map to facilitate the future study for the species of interest. Mainly due to the lack of a complete genome sequence, *M. cyclopis* is not well known and has very limited application in research. Previous phylogenetic studies solely relied on mitochondrial sequences or NRAMP1 loci^7–14^. We thus set out to sequence the *M. cyclopis* genome, in hope to facilitate its applications in disease control and the study of primate evolution.

Here, we report the *M. cyclopis* genome generated by de novo hybrid assembly^15^. This study is accomplished by using four libraries, including two Illumina paired-end (PE) short-read (SR) libraries, one Mate-Paired (MP) library, and one Nanopore long-read (LR) library, to make up a total of 115-fold coverage of the expected genome of ~ 2.9 giga base pairs (Gb). We used *M. m. mulatta* genome, Mmul_10 (Accession number: GCF_003339765.1), as a reference to facilitate the contig-to-chromosome assignment. MaSuRCA produced the best assembly metrics and high quality contigs and was used to build the *M. cyclopis* genome which contains 2,846,042,475 base pairs.

Endogenous viral elements (EVEs) represent fossil genes derived from parasitic genomes (most commonly, retroviral sequences) that integrated into the host genome during an ancient time. Most of the observed EVEs from simian virus (SIV), a retrovirus which relies on genomic insertion for replication, have undergone mutations for a long period of time. Thus, EVEs of SIV in macaques provide additional information for the study of primate evolution and medical application^16^. Previous studies have shown that many Old World Monkey species have either full length or near full-length simian endogenous retroviral sequences (SERSs) as a result of heterozygous integrations. As described by van der Kuyl and colleagues, most SERSs are of < 0.3 and 10 million years old^16^.

To further understand the geographical influence on macaque genomes, we compared the SIV *gag* (group specific antigen) and *pol* (polymerase) gene fossils in the genomes of *M. cyclopis*, which lives solely in Taiwan Island, *M. fascicularis*, which lives in the Indonesian islands, and *M. m. mulatta*, which lives in Asia continent. For the study, we employed TBLASTN^17^ to search for the EVEs of *gag* and *pol* genes. TBLASTN uses protein queries to search for the corresponding nucleotide sequences by screening all six possible reading frames to find the best alignment, whereas BLASTN uses nucleotide queries to search for similar sequences by scanning only one reading frame. As reported, TBLASTN is generally more sensitive and more accurate than BLASTN when searching for nucleotide databases^18^.

In parallel and to serve as a reference, we also searched for the EVEs of monkeypox viral genes encoding CL5 (a membrane protein), A15 (a core protein), P4a (another core protein), and D2 (a virion protein). Monkeypox virus is a double-stranded DNA virus which does not rely on genomic insertion for replication. The analysis revealed some interesting features of the *M. cyclopis* genome.

## Results

### Workflow

**Figure 1:**
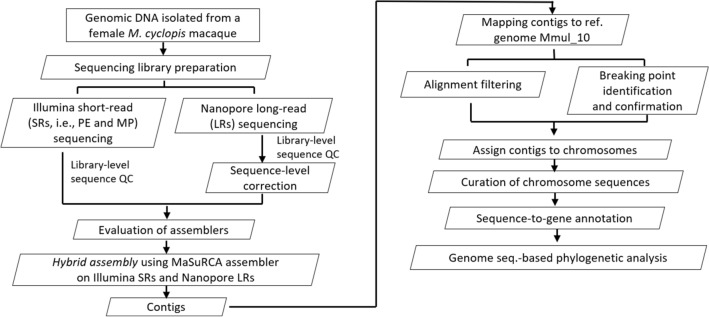
Flow diagram describing sequential steps of the assembly pipeline. Genomic DNA isolated from WBCs of a female *M. cyclopis* monkey was processed through sequencing library preparation for Illumina short-read (SR) and Nanopore long-read (LR) sequencing. Besides regular QC, Nanopore LRs were subjected to additional sequence-level correction due to higher error rate. The high-quality reads were then assembled into contigs by the MaSuRCA hybrid assembler. These contigs were then assigned to chromosomes by aligning them to the Mmul_10 reference genome. Additional curation and annotation steps were performed to produce a final draft that was subjected to genome-based phylogenetic analysis.

Workflow of *M. cyclopis* genome sequencing, assembly and annotation is depicted below (Fig. 1). Briefly, genomic DNA (gDNA) isolated from White Blood Cells (WBCs) of a female *M.cyclopis* macaque was used for genome sequencing with both Illumina and Nanopore sequencers.

Raw reads were processed to identify quality reads in each library. We used *M. m. mulatta* v10 assembly (Mmul_10) as the reference to facilitate the assignment of contigs to chromosomes. Breakpoints within contigs and gaps between contigs in each chromosome were curated and further assessed by BUSCO. Curated chromosomes were then subjected to annotation using MAKER pipeline^19^ and then used for genome-based phylogenetic analysis.

### Library statistics

A total of four libraries were sequenced from *M. cyclopis* genomic DNA for the assembly (Table 1). A 115-fold total coverage was estimated based on the *M. mulatta* genome size.

**Table 1:** Library statistics of reads produced by various sequencings.

Library		R1 (reads)	R2 (reads)	Total bases	Coverage
1	2 × 90 bp PE SRs	574,495,990	574,495,990	103,409,278,200	35X
2	2 × 150 bp PE SRs	420,437,727	420,437,727	126,972,193,554	42X
3	2 × 150 bp MPs	132,057,641	132,057,641	39,617,292,300	13X
	Total	1,126,981,358	1,127,991,358	269,998,764,054	90X
4	ONT LRs	8,551,836		76,030,640,229	25X
Total				346,029,404,283	115X

### Analysis of Nanopore long reads

Long reads produced by ONT’s GridION sequencer had a mean read length of about 8.9 kb and N50 around 11.57 kb. Mean read quality of Nanopore reads was 12.1 and over 99% of reads were above Q5 (Table S1). Nanopore LRs were further assessed by NanoPack tools^20^, to shows length distribution and length-vs-average quality distribution. Both indicated that the quality of ONT LRs fell within expected range (Fig. S1).

### Evaluation of assemblers using SRs and LRs

The performance of an assembler plays a critical role in genome assembly. The assembler pipelines tested included ABySS 2.0, SOAPdenovo2 (v2.04-r240), ALLPATHS-LG (v52488), and MaSuRCA (v3.2.8). To evaluate the effectiveness of these de novo assembly pipelines, either SRs or both SRs and LRs were utilized and the best assembler was selected based on, but not limited to, run time, number of contigs/scaffolds, N50 and the lengths of gaps.

With SRs alone, MaSuRCA emerged with the best result, as it produced the least number of scaffolds (55,553 in total) and the longest N50 (504 kb) compared to other assemblies tested (Table S2). It also produced the least number of gaps, with a total size of ~ 9 Mb comparing to other assemblies (gap size ≥ 60 Mb).

When tested with both Illumina SRs and ONT LRs using MaSuRCA for hybrid assembly. The N50 increased from 504 kb (with SRs alone) to almost 2.7 Mb (with both SRs and LRs). Moreover, the length of the longest contigs increased by ~ fourfold and the total number of contigs decreased by ~ tenfold in hybrid assembly with respect to short read assembly. Polishing of genome using short reads and PILON^21^ tool confirmed 99.24% of bases in the assembly and the N50 of the resulted assembly was slightly shorter after polishing.

We obtained 8,551,836 raw LRs from Nanopore sequencing, which were assembled to produce four different assemblies using the server in NCHC (National Center for High Performance Computing, Taiwan). Flye assembler^22^ was used at default parameters on raw as well as corrected long reads with target genome size of 3 Gb for macaques. Similarly, wtdbg2 algorithm was used for raw and polished reads to produces two more genome assemblies. Each of these tools run for about 2–3 days (Intel (R) Xeon, x86_64 GNU/Linux 64-bit processor, with 16 CPUs, 2-Threads per core and a 4 TB RAM) to obtain the results (Table S2). Total length of genome produced was 2,855,453,703 in 8615 contigs with N50 1,303,056 using Flye with raw reads. Length of longest contig was 17,917,662. We observed a decrease in N50 with corrected reads. Polishing of genome using short reads and PILON tool confirmed above 98% of bases in each of the assembly. Polished assemblies refer to nucleotide error correction that have been done using all short reads with PILON (Table S3), while unpolished assemblies refer to the raw assemblies obtained from the assembly pipeline.

We totally obtained 5065 contigs for hybrid assembly from MaSuRCA which is least among all other assembly pipelines we used that may involve short-reads or long reads only assemblies with different tools. Along with that we also observed N50 of this assembly was highest at contig level that is 2.66 Mb and only 277 contigs spanned around half the size of genome. 2,794,492,609 (98%) bases out of total 2,851,379,220 bases were present in 1971 (38.9%) contigs that were longer than 100 kb. Number of ambiguous bases or gaps in assembly were zero.

Further upon analysis using QUAST, we observed that 98.15% of reference genome was covered by *M. cyclopis* contigs. Single nucleotide mismatches and short insertions and deletions are lesser than long-reads only assemblies but slightly higher than short-reads only assemblies. BUSCO analysis revealed that assembly has similar number of BUSCOs present in MaSuRCA hybrid genome assembly (94.5%) as *M. mulatta* genome (94.4%).

We used a tailored approach that employed the contigs produced by hybrid assembly to construct chromosomes. Normally, scaffolding is performed prior to chromosome construction using the reads that could not be used in the process of contig assembly and these reads are supposed to be able to assist stitching the contigs into scaffolds. However, we used an alternative approach that includes mapping of contigs to the genome of closely related species (i.e., rhesus macaque) as reference to build the chromosomes based on alignment information. We observed that most reads were already used in building contigs. Therefore, upon scaffolding we observed a slight change in N50 (contig level N50: 2.66 Mb to scaffold level N50 2.85 Mb). We thus skipped scaffolding and proceeded to construct chromosomes directly from contigs to avoid false-positive merging of contigs.

### Evaluation of assemblers using reference and BUSCOs

We then evaluated all the assemblies produced by long-reads only, short-reads only and hybrid strategy using QUAST using Mmul_10 assembly as reference.

Among all assemblers tested, MaSuRCA produced the highest genome fraction. Compared to short reads, long reads assemblies contained significantly higher number of indels per 100 kb. On the other hand, hybrid assembly approach resulted in similar number of indels per 100 kb as short reads assemblies.

Furthermore, no additional gaps were introduced by the hybrid approach. Overall, the result suggested the hybrid approach produced better assembly than using SRs or LRs alone. From this step onwards, we retained MaSuRCA assembly as final assembly for *M. cyclopis.* Overall more than 98.3% genes and 98.3% exons were covered in the assembly. Number of partial genes were high (25.04%) *in M. cyclopis* assembly because of fragmented contigs. Number of complete genes tends to increase or decrease with N50. Also, most exons are shorter in size and hence the number of complete exons is much higher than those of genes.

BUSCO analysis with eukaryotic lineage produced similar results to *M. m. mulatta* Mmul_10 assembly. For, mammalian and vertebral lineages, MaSuRCA hybrid performed best among all other assemblies and it was also comparable to Mmul_10 assembly.

### Chromosome assembly

A total of 2395 contigs were used in chromosome construction, while contigs shorter than 10 kb or mapped within another longer contigs, were excluded. Additionally, 19 other contigs could not be used in building the draft due to complex structural variations.

When compared to the Mmul_10 genome, we found 1236 regions in Mmul_10 were missing in the *M. cyclopis* assembly. We devised a local assembly strategy to address this issue. All sequence reads were mapped to each of the Mmul_10 regions with an extension of 1 kb in either direction to identify the best matched sequences to fill the gap in the *M. cyclopis* assembly. Out of the 1236 regions, 287 were improved—either completely (e.g., Fig. S2A) or partially (e.g., Fig. S2B), and 40 regions were improved using scaffolds from Flye long read assembly. The remaining 909 regions might not present in the *M. cyclopis* genome.

Ninety-eight unplaced contigs that contained gene/exons/coding sequence (CDS) were also included in the final assembly. When compared to the initial assembly, we observed increases in number of genes, CDS and exons by 287, 2151 and 2195, respectively, in the final assembly. Lastly, BUSCO analysis revealed that the final assembly of *M. cyclopis* contains 3893 (94.9%) complete, 101 (2.5%) fragmented and 110 (2.6%) missing mammalian BUSCOs.

With these efforts, the *M. cyclopis* genome was found to comprise 2,846,042,475 base pairs. Based on this draft, we continue to perform annotation.

### Annotation

#### Repeat sequences in *M. cyclopis*

With Dfam mammalian repeats, RepeatMasker masked about 49.72% of the draft *M. cyclopis* genome (Table 2). Most repeats are SINEs (13.78%), LINEs (20.76%), LTR elements (8.99%), and interspersed repeats (47.28%). We observed high repeat content in the unplaced contigs, which could be the reason why these contigs could not be assembled into the chromosomes.

**Table 2:** Number of bases masked as repeats using Dfam mammalian repeat library and RepeatModeler2 identified repeats in *M. cyclopis*.

	Total length	Total (excluding N)	Masked with Dfam mammalian repeats	Masked with RepeatModeler2 identified repeats
chr01	221,613,023	220,994,931	112,410,065 (50.72%)	89,068,918 (40.19%)
chr02	196,042,238	193,277,993	96,268,584 (49.11%)	76,124,036 (38.83%)
chr03	184,945,174	183,274,494	90,244,834 (48.80%)	72,261,914 (39.07%)
chr04	169,525,780	167,162,861	81,774,336 (48.24%)	65,459,666 (38.61%)
chr05	187,184,648	185,212,201	93,081,134 (49.73%)	74,092,423 (39.58%)
chr06	178,587,010	176,802,830	88,273,189 (49.43%)	69,653,005 (39.00%)
chr07	168,997,397	165,390,027	82,641,896 (48.90%)	66,111,160 (39.12%)
chr08	144,803,349	141,891,607	71,323,313 (49.26%)	54,800,658 (37.84%)
chr09	131,916,780	130,825,946	63,734,884 (48.31%)	50,788,285 (38.50%)
chr10	98,080,139	94,988,555	47,915,172 (48.85%)	37,749,017 (38.49%)
chr11	136,824,654	130,756,091	67,363,374 (49.23%)	53,417,348 (39.04%)
chr12	129,984,955	128,084,127	61,049,974 (46.97%)	48,346,610 (37.19%)
chr13	109,383,283	107,259,873	52,898,341 (48.36%)	41,079,629 (37.56%)
chr14	127,240,383	125,484,249	62,801,306 (49.36%)	48,638,064 (38.23%)
chr15	111,340,568	108,375,977	54,531,030 (48.98%)	42,455,782 (38.13%)
chr16	79,137,903	77,178,102	38,800,692 (49.03%)	31,401,587 (39.68%)
chr17	93,296,330	92,380,788	43,503,816 (46.63%)	34,406,182 (36.88%)
chr18	74,421,989	72,824,267	34,282,577 (46.07%)	26,290,867 (35.33%)
chr19	59,178,552	54,338,847	32,750,473 (55.34%)	26,875,186 (45.41%)
chr20	76,953,612	75,498,354	38,728,815 (50.33%)	30,349,703 (39.44%)
chrX	152,979,943	151,613,795	93,150,839 (60.89%)	77,281,821 (50.52%)
Unplaced	13,604,765	13,604,465	7,544,110 (55.45%)	6,401,348 (47.05%)
Total	2,846,042,475	2,797,220,380	1,415,072,754 (49.72%)	1,123,053,209 (39.46%)

On the other hand, RepeatModeler2 predicted 937 repeat families in *M. cyclopis*, which covered about 39.46% of *M. cyclopis* genome. Similarly, most repeats are SINEs (12.36%), LINEs (15.54%), LTR elements (7.18%), and interspersed repeats (37.71%). Of the repeat families identified, 709 repeat families contributed to novel 32,754 regions (5,240,159 bases) that could not be identified by Dfam mammalian repeats. This information is highly valuable to enhance the existing annotation of mammalian repeats.

#### Genome annotation

**Figure 2:**
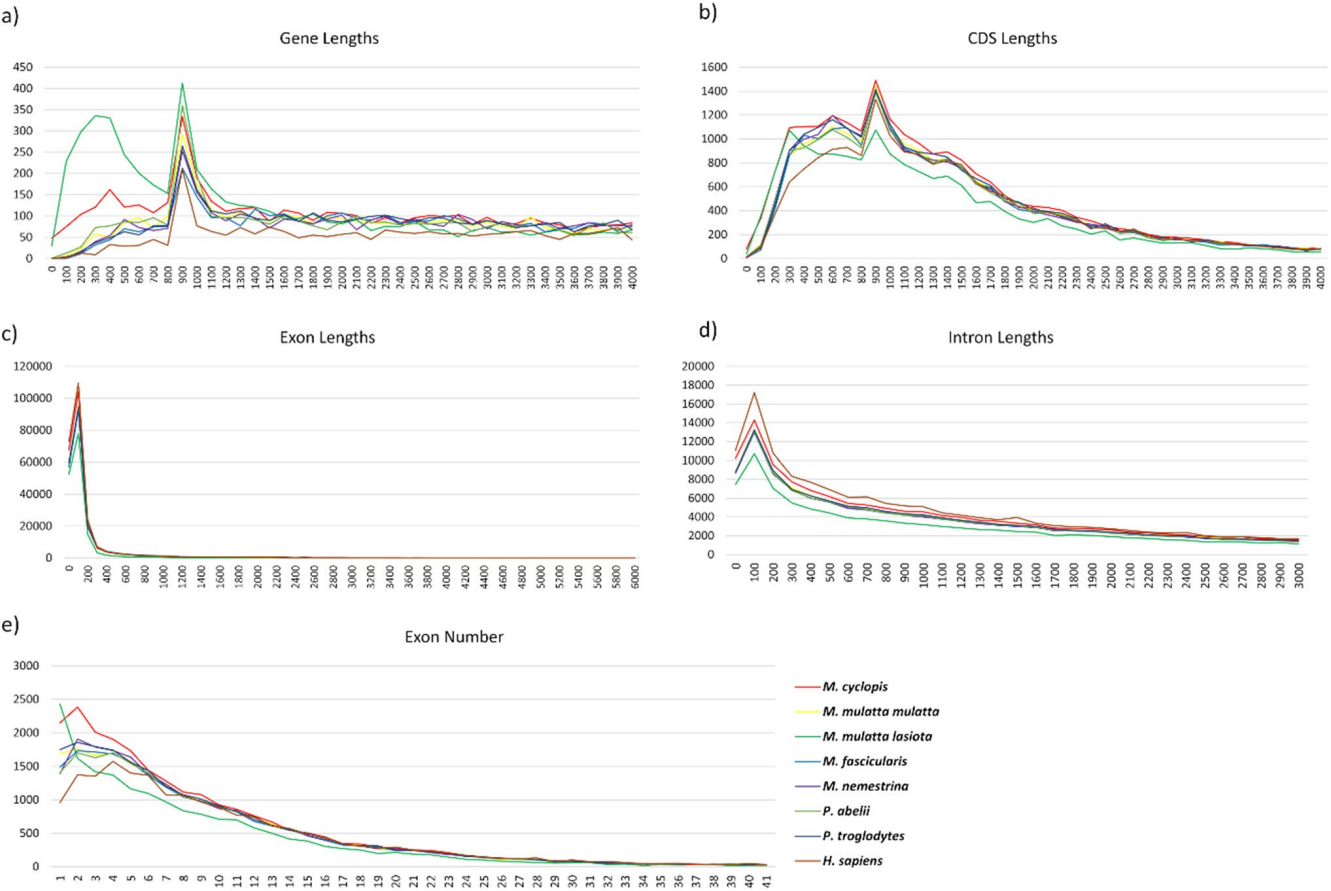
Whole-genome comparison of basic genomic features among different primate species. Gene lengths (**a**), CDS lengths (**b**), exon lengths (**c**), intron lengths (**d**) and number of exons (**e**) of five macaque species (i.e., *M. cyclopis*, *M. m. mulatta*, *M. mulatta lasiota, M. fascicularis*, and *M. nemestrina*), *Pongo abelii* (Sumatran orangutan), *Pan troglodytes* (chimpanzee) and *H. sapiens* (humans) are compared to depict the differences in the structure of protein-coding genes. X axis represents the length in bp; Y axis represent the corresponding numbers.

We used 86,716 transcripts and 67,976 proteins from *M. mulatta* genomes as the input for MAKER to annotate the *M. cyclopis* genome. Genes in the *M. cyclopis* genome were predicted based on homology-based method and *ab-initio* method incorporated in the MAKER pipeline. The analysis revealed 23,462 protein-coding genes with 716,231 exons and 59,484 transcripts. By comparing the genome annotation of seven other primate species, we observed a high degree of similarity in gene, exon, CDS and intron lengths of *M. cyclopis* with that of other species (Fig. 2). Several non-coding RNA species have been identified including 609 miRNAs, 600 tRNAs, 4217 lncRNAs and 580 rRNAs. Genomic locations of these non-coding genes are provided in the supplementary files (Supplementary information).

#### Analysis of genomic translocations

**Figure 3:**
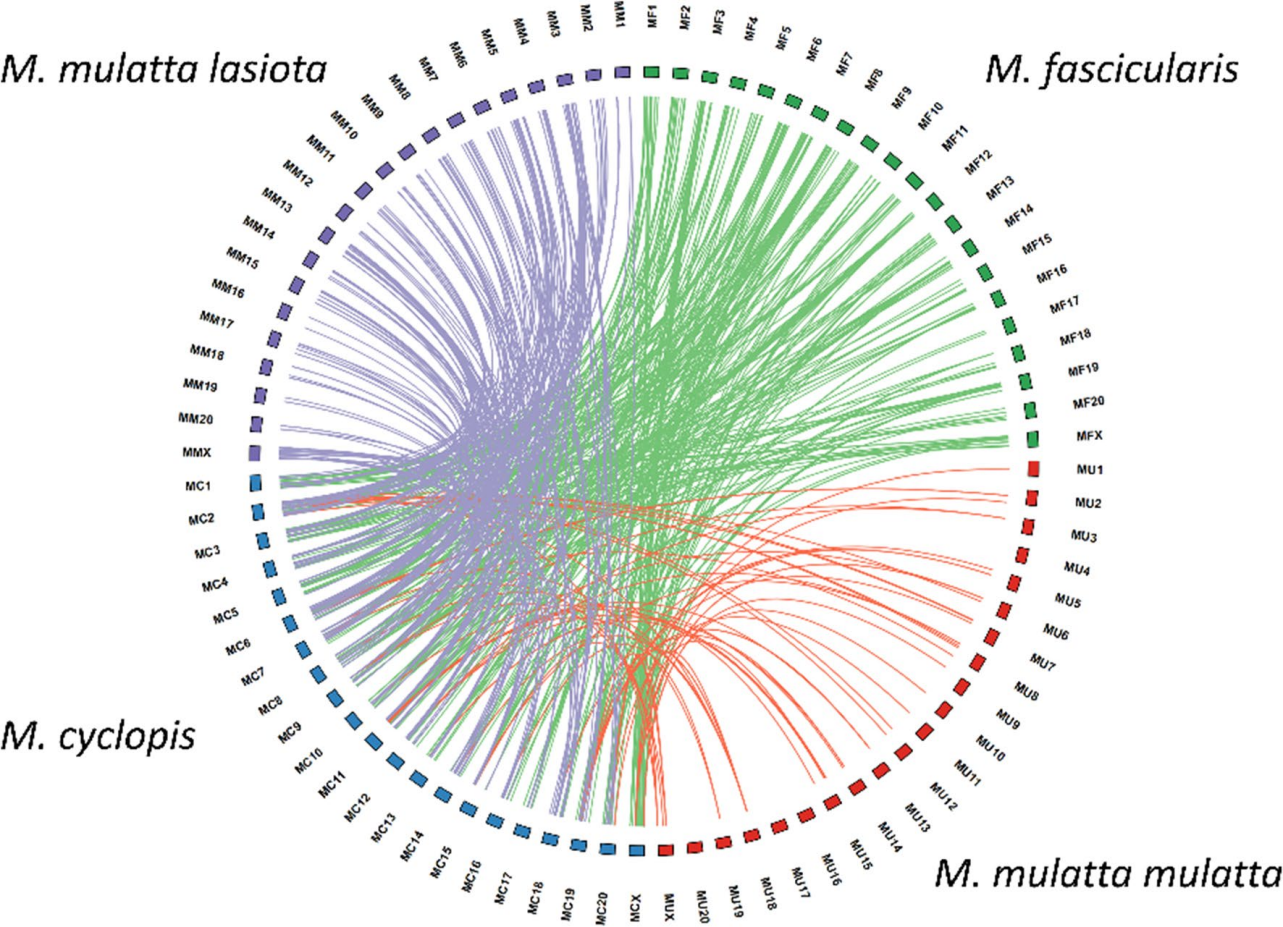
Circular plot showing translocations of chromosomal fragments with sizes ≥ 10 kb between *M. cyclopis* and *M. mulatta* or between *M. cyclopis* and *M. fascicularis*. Chromosomes are radially aligned in a circle (red, *M. m. mulatta*; green, *M. fascicularis*; blue, *M. cyclopis*; purple, *M. mulatta lasiota* followed by chromosome number). Arcs within the circle indicate interchromosomal translocations of fragments with sizes ≥ 10 kb. There are 358 translocations between *M. cyclopis* and *M. fascicularis* (green), 512 translocations between *M. cyclopis* and *M. mulatta lasiota* (purple) and 60 translocations between *M. cyclopis* and *M. m. mulatta* (red).

Alterations in genomic sequences among *M. cyclopis*, *M. m. mulatta*, *M. mulatta lasiota* and *M. fascicularis* were depicted with Circular plot (Fig. 3). We observed significant degree of genomic translocations between *M. cyclopis* and *M. fascicularis*, much higher than that between *M. cyclopis* and *M. m. mulatta*. This result is of high degree of concordance with previous reports^8^, showing that with respect to *M. cyclopis*, *M. fascicularis* is more distal in evolution than *M. m. mulatta*.

#### Genome-based phylogenetic analysis

**Figure 4:**
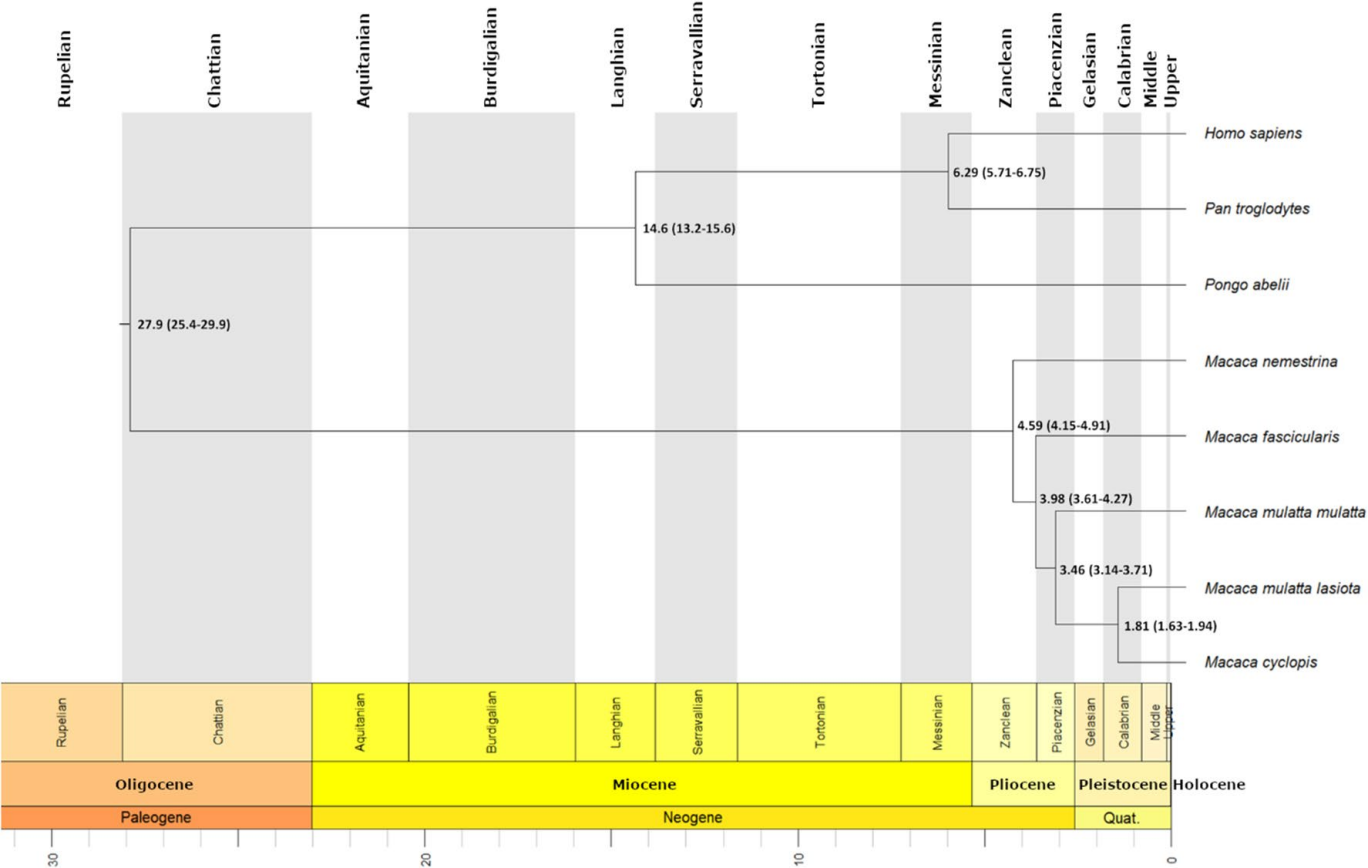
Phylogenetic tree of eight different species with divergence time. Nodes are labelled with their putative divergence time in million years and the range with 95% confidence interval is provided in parenthesis.

The complete *M. cyclopis* genome allowed us to perform a phylogenetic analysis based on amino acid sequences of proteins derived from the assembled chromosomes. Phylogenetic tree was constructed using single copy orthologs in *M. cyclopis* and seven other primate species (i.e., *H. sapiens* (GCF_000001405.39), *Pan troglodytes* (GCF_002880755.1)*, Pongo abelii* (GCF_002880775.1), *Macaca nemestrina* (GCF_000956065.1), *Macaca fascicularis* (GCF_000364345.1), *Macaca m. mulatta* (GCF_003339765.1) and *Macaca m. lasiota* (GCF_000230795.1) to calculate the divergence time for *M. cyclopis* (Fig. 4). Further analysis of the results indicated that the Formosan macaque diverged from Chinese rhesus macaque (*M. m. lasiota*) about 1.8 million years ago.

The topology of our tree is consistent with those based on previous phylogenetic studies of primate species and estimates of dates of inter-species divergence. These include studies of electrophoretically defined protein coding loci, mtDNA RFLPs, sequences and whole genomes, STRs, SNPs and nuclear gene sequences. The first split in our tree separates the families Hominidae and Cercopithecidae which diverged from each other approximately 29 million years ago (mya)^23,24^. Of living species of Hominidae, *Pongo* is the most distant, as in our own tree, having separated from a clade shared with *Pan*, *Homo* and *Gorilla* several million years prior to the split between *Pan*/*Homo* and *Gorilla*, between 8 and 10 mya, and that between *Pan* and *Homo*, some 6.5 mya^23–25^. Genus *Macaca* includes approximately 20 species which represent what most scholars^26,27^ regard as four monophyletic groups of species. One of these four species groups, the silenus group, is that from which all other groups derive, and includes *M. nemestrina,* the oldest and most divergent cercopithecoid species in our tree. Another of the four groups, the fascicularis group, includes the remaining three cercopithecoid species (*M. fascicularis*, *M. mulatta* and *M. cyclopis*) comprising, with *M. fuscata* of Japan, the fascicularis group of macaque species^28^. *M. mulatta* is believed to have diverged from a fascicularis-like ancestor as early as 3.24 mya^29^ after the split of *M. nemestrina*, the earliest split from *M. sylvannus*, the ancestor of all macaques, about 7 mya. *M. cyclopis* is believed to have derived from a *M. mulatta* population that colonized Taiwan from southern China and Vietnam between 0.38 and 0.44 mya^4^. Therefore, our tree comports perfectly with previous phylogenetic studies.

#### SIV *gag* and *pol* EVEs were detected in *M. fascicularis*, *M. m. mulatta* and *M. cyclopis*, while none of these macaques harbour EVE-like sequences of the monkeypox viral genes tested

Fossil gene analysis by TBLASTN revealed the presence of EVEs of SIV *gap* and *pol* genes in all macaques tested, including *M. fascicularis, M.* m. *mulatta* and *M. cyclopis*. Among these three species, *M. fascicularis* genome harbours the highest number of both genes (56 sites for *gag*; 76 sites for *pol*), followed by *M. m. mulatta* genome (44 sites for *gag*; 64 sites for *pol*), and then the *M. cyclopis* genome (24 sites for *gag*; 23 sites for *pol*) (Table S4). On the other hand, all monkeypox genes tested showed no significant matches by TBLASTN under the same TBLASTN parameters. Taken together, the above fossil gene search indicates that *M. cyclopis* is unique in both number and distribution pattern compared to that of *M. fascicularis* and *M. mulatta*. In contrast, fossil gene analysis using same parameters showed no significant EVEs for all the monkeypox genes tested across all these macaques.

## Discussion

Whole genome sequencing enhances medical research and evolutionary study. Previous studies showed that macaques shared a common ancestor with human about 25–32 mya and diverged from each other about 5–6 mya^30,31^. Here, whole-genome phylogenetic analysis suggests that *M. cyclopis* diverged from *M. m. lasiota* about 1.8 mya, which was much more recent compared to the onset of divergence of macaque species.

By providing the genome sequence of *M. cyclopis*, we add an additional piece of information for the study of primate evolution. This sequence is also reported in NCBI as reference enabling researchers to use it in genomics-based studies of Taiwanese macaque. List of variations in *M. cyclopis* provided will also aid the studies on genetic differences that are responsible for the phenotypic diversity in macaques. Additionally, this work also opens up the opportunity for the usage of *M. cyclopis* as an animal model for medical research.

Previous phylogenetic studies indicated that *M. cyclopis* descended from Chinese monkey *M. m. lasiota*^8^. With a completed *M. cyclopis* genome, we further demonstrate that the distributions of *gag* and *pol* EVEs in *M. cyclopis* is relatively low in number, but with higher uniformity in distribution patterns, readily distinguishable from that of *M. fascicularis* and *M. m. mulatta*. The uniqueness, which might have resulted from geographical isolation through the evolutionary process, suggests that *M. cyclopis* is a macaque particularly useful in medical research for the study of infectious diseases. The higher complexity of SIV fossil gene distribution pattern found in *M. m. mulatta* and *M. fascicularis*, as compared to that of *M. cyclopis*, is likely to result from the fact that these two macaques live in the wide-open continental area and islands in close proximity. As horizontal gene transfer is an important factor for gene transfer of SIV sequences among macaques^32^, such habitats provide great opportunities for cross-species and cross-organism gene transfer, not only for SIV, but also for other retroviruses as well, causing the increase in their genome complexity. When used in medical research, the genome complexity would more or less hinder the downstream analyses such as transcriptome analysis, transcription factor binding site analysis, and many others. On the other hand, geographical isolation of *M. cyclopis* in Taiwan Island limited the cross-species gene transfer so to result in the relative simplicity of the *M. cyclopis* genome, making *M. cyclopis* an ideal animal, or even better than *M. mulatta*, for biomedical research.

In terms of the usage of short reads and long reads, short reads are high in accuracy but each covers only very limited genomic range (≤ 300 bp for PE reads). On the other hand, although long reads are lower in quality but each covers a longer genomic distance. With a combination of SRs and LRs, hybrid sequencing has been commonly employed for the assembly of large genomes. To improve the accuracy, all LRs were corrected by SRs prior to hybrid assembly. With over 100-fold coverage and the usage of a closely related species, *M. mulatta*, as the reference, we intended to reach a fair degree of accuracy. Transcriptome or exome sequencing data should be able to validate the results. However, we are currently short of these types of data.

In summary, this work aims to set a tone for *M. cyclopis*-related studies and applications in the future. Results of this work seem to agree well with the previous reports. In addition, it also provides additional valuable information for future investigations. To move one step further, detailed SNP analysis will be helpful.

## Methods

### Ethical statement

The animal handling procedure and experimental protocols of this study fully complied with the guidelines of IACUC (Institutional Animal Care and Use Committee) and NPUST (National Pingtung University of Science and Technology) (Please see The Guide for Animal Use and Care under http://lac.npust.edu.tw/files/15-1112-30104,c4232-1.php?Lang=zh-tw) and was approved by Pingtung Rescue Center for Endangered Wild Animals (PTRC) at NPUST. All people involved in handling the animal were well trained. Moreover, this study is also in accordance with ARRIVE guidelines, and no human subject was involved in this study.

### Source of DNA

A captive female Formosan macaque in the Pingtung Rescue Center for Endangered Wild Animals (PRCEWA) of National Pingtung University of Science and Technology (NPUST) was used for the study. She (NPUST ID: 10D7) was about 22-year old when her blood was first collected after anesthesia following NPUST guidelines. Genomic DNA from white blood cells (WBCs) was isolated right after blood collection and stored at − 20 °C before use.

### Short-read sequencing and data processing

To prepare sequencing library for SR sequencing, DNA was randomly sheared by sonication and 300–500 bp fraction was isolated by gel excision and used for Illumina paired-end (PE) following the instructions provided by the manufacturers. SR libraries used in this work include (1) 2 × 90 bp PE SR library, sequenced by HiSeq 2000 (BGI); (2) 2 × 150 bp PE SR library, sequenced by HiSeq X Ten (Macrogen Inc.); and (3) 2 × 150 bp mate-pair (MP) library with insert size of ~ 3 kb was also prepared and sequenced (BGI).

SR raw reads were processed by the following procedure. Raw reads were examined with FastQC^33^ for base quality and the presence of adapters. AdapterRemoval tool^34,35^ was then used to remove the adapter sequences if present. Terminal ambiguous bases were trimmed off by Ambiguity trimming module of NGS QC Toolkit, and low quality reads were filtered out using IlluQC module of NGS QC Toolkit^36^. Reads >  = 100 bp were selected by PRINSEQ software^37^. Mapping to reference genome was performed by BWA^38^. The insert size of MP reads was estimated by BamTools-based script and reads with insert sizes of about 2600 (bp) +/− 30% were selected and used in assembly.

### Nanopore long-read sequencing and data processing

DNA samples isolated from WBCs of the same female monkey were also sequenced by Health GeneTech (Taiwan) using Oxford Nanopore GridION sequencer. Three micrograms of high molecular weight genomic DNA was sheared to 8–12 kb fragments by G-tube (Covaris) based on the protocol of the company. DNA was subsequently size-selected using 0.45X volume of Ampure XP (Beckman Coulter) and eluted in 48 µl nuclease-free water. Sequencing library was prepared using ONT (Oxford Nanopore Technologies) 1D ligation sequencing kit (SQK-LSK109) following manufacturer’s protocol. Briefly, FFPE DNA repair and end repair process was completed by NEBNext Ultra II End Repair/dA-Tailing Module (New England Biolabs). Blunt/TA Ligase Master Mix (New England Biolabs) was used to ligate the sequencing adapters to library. The GridION sequencing was performed using a FLO- MIN106D flow cell till all pores were exhausted. A total of 6 flow cells were used in sequencing to generate a total of 75 billion bases. NanoPlot tool was used to generate a summary for ONT long-reads^20^.

Nanopore reads were corrected with short reads using proovread software under default parameters^39^. Untrimmed reads from the output were used as corrected reads for further steps of assembly.

### Selection of MaSuRCA for hybrid assembly

A number of assemblers, including ABySS 2.0^40,41^, ALLPATHS-LG^42–44^, SOAPdenovo2^45,46^, MaSuRCA^47^, FLYE 2.4.2^22^, wtdbg2^48^ and Spades^49^, were evaluated and their performances were compared with various metrics commonly used in genome assembly. All these pipelines are capable of integrating multiple libraries of variable read lengths. In our study, machine with Intel (R) Xeon, x86_64 GNU/Linux 64-bit processor, with 16 CPUs, 2-Threads per core and 4 TB RAM was used for genome assembly with different pipelines.

A hybrid assembly was generated by MaSuRCA 3.28 on the machine using three Illumina libraries (two SR libraries and the MP library) together with a ONT LR library (configuration file was changed with “CA_PARAMETERS = cgwErrorRate = 0.15 merylMemory = 2 GB ovlStoreMemory = 2 GB” and other parameters set to default). Contigs produced by hybrid assembly were polished by mapping short reads of the 2 × 150 PE and 2 × 90 PE libraries using BWA. Errors were subsequently corrected by PILON based on the consensus generated from reads mapped to the location^21^.

### Evaluation and correction of contig misassembly prior to chromosome assembly

Alignment filtration involves exclusion of contigs, if it mapped completely within the range of another contig or it was shorter than 10 kb. Breakpoints in contigs were identified, if (1) mapped in one alignment to one chromosome; (2) mapped in multiple segments far from each other in same chromosome; (3) mapped in multiple alignments on different chromosomes. These breakpoints were confirmed from short-reads alignment of assembly and contigs were broken, if short reads are not mapped at breakpoints in concordant manner or there were no short reads are mapped at breakpoints. Contigs were placed at a gap proportional to their mapping distance on reference chromosome and merged if their mapping is overlapping on the reference.

Contigs from hybrid assembly were further evaluated by the following steps. We mapped the reads from each library using BWA on polished assembly as reference to estimate the correctness of assembly. (1) Contigs were mapped to the reference Mmul_10 using minimap2^50^. (2) All alignments of a contig that were contained within a larger alignment of another contigs were removed. (3) Contigs that were mapped (a) with single map on one chromosome, b) with multiple hits on one chromosome, (c) with single/multiple hits to multiple chromosomes were identified. (4) Contigs with multiple hits were checked if the hits were in consecutive to each other, if not, then their break point were checked for the read support at breakpoint locations. (5) Breakpoints within contigs that were covered by concordant reads were not broken and rest were broken since they were not covered by any concordant reads. (6) Contigs were stitched to one another based on their alignment order with respect to reference. Same length of gap was inserted between two contigs if they are non-overlapping. In case of overlap in between pair of contigs they were merged together. (7) Contigs mapped in the regions of complex structure variations were visualized using IGV and their breakpoints were recorded. Using the support from short reads insert-size we estimated the order of contigs manually. (8) Regions from Mmul_10 chromosomes that were not covered by contigs were recorded from previous step of chromosome generation. All the reads mapped in these regions including 1000 bases upstream and downstream of uncovered region were retrieved from whole genome reads files for each library. These reads were used for local assembly of uncovered regions. Contigs produced from this procedure were mapped to *M. cyclopi*s draft chromosomes to find the anchors for new contigs and to patch the gap regions in *M. cyclopi*s genome. (9) We next used contigs longer than 10 kb and searched if these contigs may have sequence similarity upstream or downstream to the remaining gaps. If found similarity these contigs were merged into the genome to reduce the span of gaps in the assembly. Remaining gaps were filled with the long-reads assembly produced using Flye assembler with the similar strategy as shown above. Flye scaffolds that spanned throughout the gap on the reference genome region were used to fill in the gap in the *M. cyclopis* genome.

### Further curation of chromosome assemblies

Chromosomes were evaluated with QUAST on default parameters with *M. mulatta* assembly Mmul_10 as reference^51^. We used gene and exon information of Mmul_10 for feature based evaluation separately to evaluate whether the genes/exons/CDS have been covered completely or partially in an assembly.

After evaluation using QUAST, we analysed the genomic features obtained. We estimated the number of missing genes, exons and CDS in the chromosome level assembly and tried to recover those contigs which could not be stitched into the chromosomes. We also found missing genes, exons and CDS in the locally assembled contigs. These contigs which might have information about gene features were retained as unplaced contigs in the final assembly. Further we analysed whether these genes were not present in the genome or could not be detected due to technical issue.

Lastly, we used BUSCO tool to estimate the number of BUSCOs present in each assembly. Three different lineages (eukaryotic, mammalian and vertebral) of BUSCO were searched in each assembly^52^.

### Repeat masking and identification

RepeatMasker version 4.1.5 (http://www.repeatmasker.org) were used to mask the draft genome of *M. cyclopis*, using the curated families of Dfam^53^ (version 3.7). We then used RepeatModeler2^54^ to predict repeats from *M. cyclopis* using RECON^55^ and RepeatScout^56^ with default parameters. The output of RepeatModeler2 was then used to mask *M. cyclopis* genome to identify potential de novo repeat regions.

### Genome annotation

For genome annotation we employed GEAN^57^ to annotate the genome. We used *M. mulatta* transcriptome assembly and protein sequences for initial genome annotation. Annotations from GEAN were further processed with MAKER v2.31.8 pipeline^58^. We then used SNAP, Genemark-ES and AUGUSTUS to predict the gene and exonic boundries^59–61^. Annotation of seven species were analysed including *M. cyclopis*, *M. m. mulatta*, *M. fascicularis, M. nemestrina*, *Pongo abeli*, *Pan troglodytes* and *Homo sapiens* and gene, CDS, exon and intron lengths were compared. We used a number of software tools to identify non-coding RNAs. These include tRNAscan-SE 2.0^62,63^ for tRNAs, LncFinder v1.1.4^64^ for lncRNAs, RNAmmer v1.2^65^ for rRNAs and in-house scripts (based on BLAST 2.9.0+) for miRNA. Micro-RNA sequences of *M. m. mulatta* reference genome were extracted and used to search homologs in *M. cyclopis* genome by allowing a maximum of two mismatches.

### Evolutionary study among key primate species with the assembled *M. cyclopis* genome

To identify the gene families in Taiwanese macaque, we retrieved the protein-coding genes from NCBI for seven other primate species (*Homo sapiens*, *Pan troglodytes*, *Pongo abeli*, *M. nemestrina*, *M. fascicularis, M. m. mulatta* and *M. m. lasiota*). The longest isoforms were used as the representative genes. Orthomcl v2.0.9 pipeline was used to identify the gene families in seven species at default parameters. Protein sequences of single copy orthologs present in all eight species were used for the multiple sequence alignment using MUSCLE v3.8.1551 at default parameters and Gblocks v0.91b^66^ was used to remove the poorly aligned regions. These sequences were concatenated to build a phylogenetic tree using RAxML v8.2.12^67^. Further MrBayes v3.2.7a^68^ was used to maximum-likelihood phylogenetic inference using 10 million generations by providing an estimate of tree age about 28.8 mya taken from TimeTree database^69^. “Strap” package^70^ in R was used to plot the phylogenetic tree.

### Fossil gene analysis

In an attempt to further understand the potential differences in genome structure between *M. cyclopis* and its macaque relatives with close geographic proximity, we employed TBLASTN to identify the genomic distribution patterns of SIV *gag* and *pol* EVEs in *M. fascicularis*, *M. m. mulatta* and *M. cyclopis*. In parallel, monkeypox virus genes *OPG099* (encoding CL5 membrane protein)*, OPG136* (encoding A15 core protein)*, OPG114* (encoding P4a core protein) and *OPG142* (encoding D2 virion protein) were also tested for their corresponding EVEs.

All sequences for the fossil gene tested were downloaded from NCBI. These include the *M. m. mulatta* genome assembly Mmul_10 (ac. no.: GCF_003339765.1), *M. fascicularis* genome assembly MFA1912RKSv2 (ac. no.: GCF_012559485.2). SIV gene: *gag* (ac. no.: AAC97563.1) and *pol* (ac. no.: AAC97565.1). Monkeypox genes: *OPG099* (ac. no.: YP_010377081.1)*, OPG136* (ac. no.: YP_010377124.1)*, OPG114* (ac. no.: YP_010377118.1) and *OPG142* (ac. no.: YP_010377096.1).

As parameter setting, TBLASTN alignments with a query coverage of more than 50% of the amino acid sequence and an E-value of less than 0.05 were considered as significant. The chromosomal locations of the EVEs sites in the tested macaques were recorded.

## Supplementary Information

Supplementary Information.

## Data Availability

This Whole Genome Shotgun project has been deposited at DDBJ/ENA/GenBank under the accession JAHZQV000000000. The version described in this paper is version JAHZQV010000000. The whole genome sequencing data of *M. cyclopis* including two PE short reads, one MP short reads and ONT long reads were submitted to NCBI database with accession numbers Bioproject accession PRJNA559050 and Biosample accession SAMN12512033.
