# Supplementary material for: Genome sequencing and application of Taiwanese macaque Macaca cyclopis
Source: Sci Rep. 2023 Jul 17;13:11545. doi: 10.1038/s41598-023-38402-4 (PMC10352370; doi:10.1038/s41598-023-38402-4)
Supplement: Supplementary file 1 — Supplementary Information. [file 41598_2023_38402_MOESM1_ESM.docx]

**Supplementary information**

**Supplementary Figures**

| A  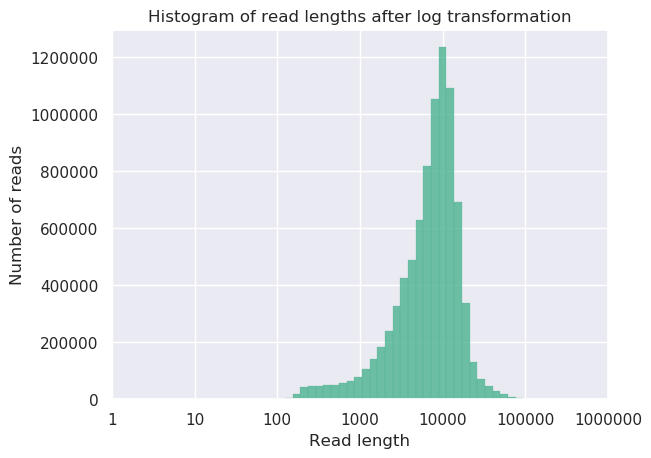 | B  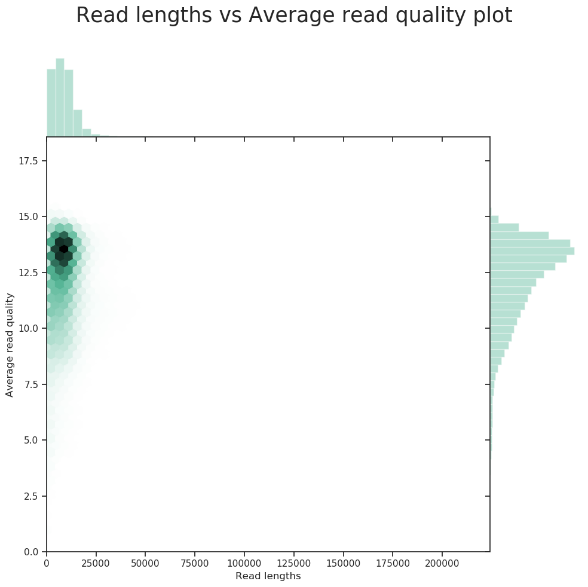 |
| --- | --- |

**Figure S1. Assessment of Nanopore long reads with Nanopack tools.** **A.** Plot of read length distribution. **B.** Plot of average read quality vs. read length.


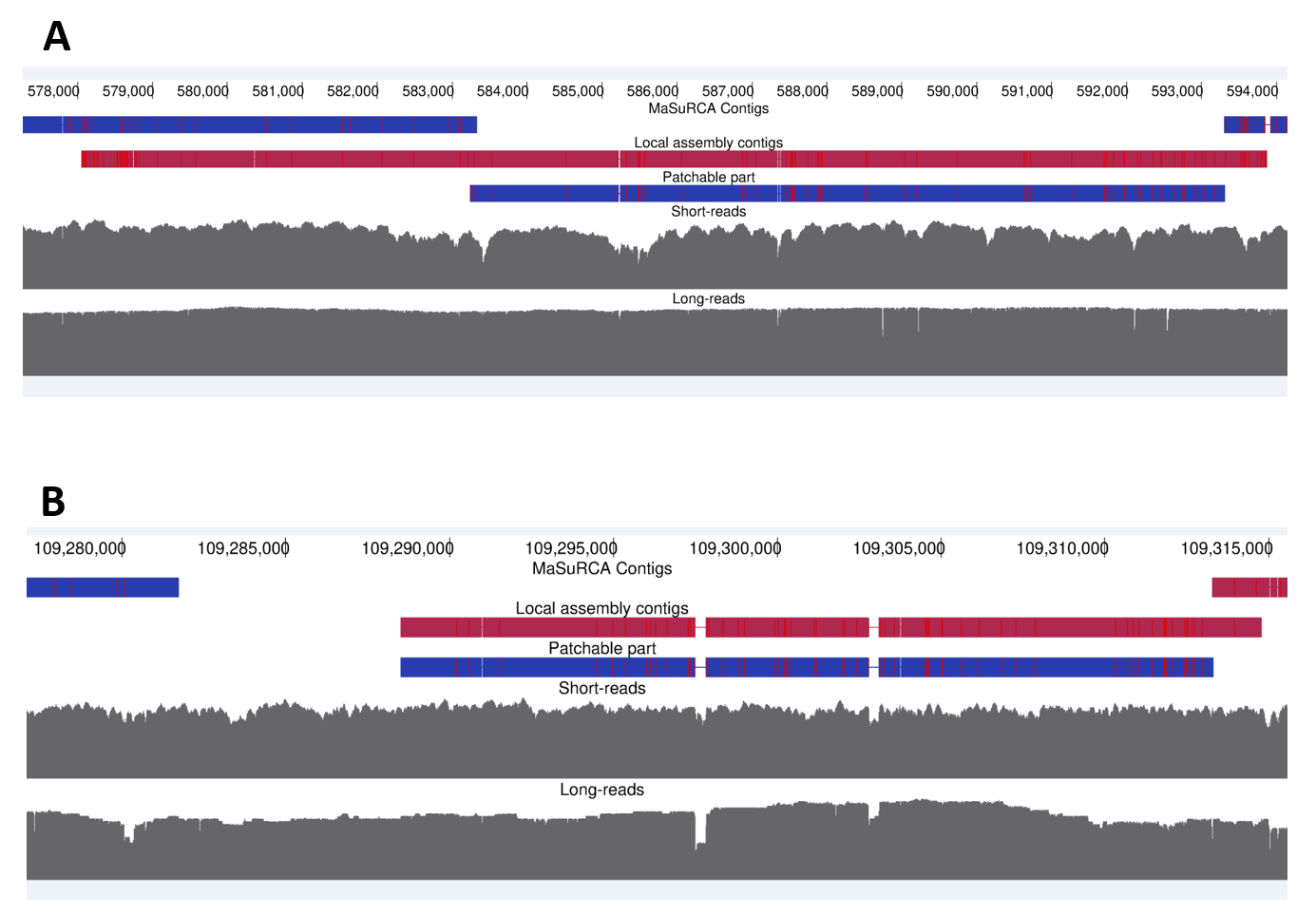


**Figure S2. Gap filling with consensus sequences produced from local assembly.** **A:** completely filled region in chromosome. **B:** Partially filled region in chromosome. Data displayed by UCSC genome browser.

**Supplementary Tables**

**Table S1. General summary of Nanopore long reads**

| Number of reads | 8,551,836 |  |
| --- | --- | --- |
| Total bases | 76,030,640,229 |  |
| Mean read length | 8,890.60 |  |
| Median read length | 8,008 |  |
| Mean read quality | 12.1 |  |
| Median read quality | 12.6 |  |
| Read length N50 | 11,570 |  |
|  |  |  |
| Quality value distribution | | |
| Q-value | Number of reads | Percentage |
| ≥ Q5 | 8,479,034 | 99.10% |
| ≥ Q7 | 8,338,492 | 97.50% |
| ≥ Q10 | 7,254,860 | 84.80% |
| ≥ Q12 | 5,190,105 | 60.70% |
| ≥ Q15 | 25,585 | 0.30% |

**Table S2. Comparison between assemblers prior to polishing**

| Fundamental data | | | | | |
| --- | --- | --- | --- | --- | --- |
| Assembly | Total Length | No. of Scaffolds | Longest Scaffold | N50 |  |
| MaSuRCA_hybrid | 2,851,379,220 | 5,065 | 15,033,544 | 2,662,167 |  |
| Flye_raw_ont | 2,855,453,703 | 8,615 | 17,917,662 | 1,303,056 |  |
| Flye_cor_ont | 2,804,382,095 | 7,357 | 15,201,322 | 1,122,863 |  |
| Wtdbg2_raw_ont | 2,772,902,571 | 10,165 | 11,019,242 | 1,405,467 |  |
| Wtdbg2_cor_ont | 2,805,071,358 | 14,479 | 7,107,886 | 843,585 |  |
| MaSuRCA | 2,801,815,577 | 55,553 | 3,705,697 | 506,040 |  |
| ALLPATHS | 2,728,019,337 | 56,467 | 1,522,448 | 143,443 |  |
| ABySS | 3,259,619,136 | 6,019,194 | 989,977 | 72,341 |  |
| SOAP | 3,875,873,933 | 6,898,267 | 2,017,445 | 46,388 |  |
| Genome fraction, duplication ratio and number of N’s, mismatches and indels in assembly using SRs and LRs. (updated 21-08) | | | | |  |
| Assembly | Genome fraction (%) | Duplication ratio | #N/ 100 kb | #mismatches/ 100 kb | #indel/ 100 kb |
| MaSuRCA_hybrid | 98.152 | 1.019 | 0 | 393.02 | 89.55 |
| Flye_raw_ont | 97.337 | 1.015 | 0.07 | 389.79 | 135.69 |
| Flye_cor_ont | 97.009 | 1.014 | 0.03 | 365.67 | 128.19 |
| wtdbg2_raw_ont | 95.407 | 1.005 | 0 | 416.97 | 184.91 |
| wtdbg2_cor_ont | 95.586 | 1.019 | 0 | 409.89 | 148.5 |
| MaSuRCA | 97.587 | 1.01 | 273.91 | 358.19 | 76.19 |
| ALLPATHS | 93.83 | 1.03 | 2318.66 | 340.22 | 70.23 |

**Table S3. Comparison between assemblers after polishing**

| Polished assembly | Total Length | No. of contigs | Longest contigs | N50 |
| --- | --- | --- | --- | --- |
| MaSuRCA_hybrid | 2,850,177,397 | 5,065 | 15,029,858 | 2,662,041 |
| Flye_raw_ont | 2,845,640,540 | 8,615 | 17,830,466 | 1,298,086 |
| Flye_cor_ont | 2,792,382,565 | 7,357 | 15,116,357 | 1,117,872 |
| wtdbg2_raw_ont | 2,788,652,410 | 10,165 | 11,070,713 | 1,414,097 |
| wtdbg2_cor_ont | 2,806,744,540 | 14,479 | 7,110,562 | 845,448 |
| MaSuRCA | 2,801,250,985 | 55,553 | 3,704,626 | 506,389 |
| ALLPATHS | 2,728,054,850 | 56,467 | 1,522,965 | 143,452 |
|  |  |  |  |  |
| Comparison of polished assemblers | |  |  |  |
| Genes covered |  |  |  |  |
| Polished assembly | #Complete (%) | #Partial (%) | #Missing (%) |  |
| MaSuRCA_hybrid | 28,721 (73.26) | 9,817 (25.04) | 665 (1.7) |  |
| FLYE_raw_ont | 27,942 (71.28) | 10,597 (27.03) | 664 (1.69) |  |
| FLYE_cor_ont | 28,184 (71.89) | 10,557 (26.93) | 462 (1.18) |  |
| WTDBG2_raw_ont | 26,072 (66.51) | 10,924 (27.87) | 2,207 (5.63) |  |
| WTDBG2_cor_ont | 26,159 (66.73) | 11,117 (28.36) | 1,927 (4.92) |  |
| MaSuRCA | 26,442 (67.45) | 12,337 (31.47) | 424 (1.08) |  |
| ALLPATHS | 21,513 (54.88) | 16,679 (42.55) | 1,011 (2.58) |  |
| Exons covered |  |  |  |  |
| Polished assembly | #Complete (%) | #Partial (%) | #Missing (%) |  |
| MaSuRCA_hybrid | 241,339 (98.1) | 489 (0.2) | 4,186 (1.7) |  |
| FLYE_raw_ont | 240,962 (97.95) | 625 (0.25) | 4,427 (1.8) |  |
| FLYE_cor_ont | 242,726 (98.66) | 594 (0.24) | 2,694 (1.1) |  |
| WTDBG2_raw_ont | 231,407 (94.06) | 856 (0.35) | 13,751 (5.59) |  |
| WTDBG2_cor_ont | 232,328 (94.44) | 862 (0.35) | 12,824 (5.21) |  |
| MaSuRCA | 242,370 (98.52) | 860 (0.35) | 2,784 (1.13) |  |
| ALLPATHS | 235,902 (95.89) | 2,203 (0.9) | 7,909 (3.21) |  |
|  |  |  |  |  |
| #genes exons and CDS identified by polished MaSuRCA | | |  |  |
|  | Assembly level | Complete | Partial | missing |
| Gene | Contigs | 28,721 (73.26) | 9,817 (25.04) | 665 (1.7) |
|  | Chromosomes | 28,725 (73.27) | 9,829 (25.07) | 649 (1.66) |
|  | Polished chromosomes | 28,744 (73.32) | 9,817 (25.04) | 642 (1.64) |
|  | Final assembly | 28,919 (73.77) | 9,859 (25.15) | 425 (1.08) |
| CDS | Contigs | 217,328 (98.28) | 92 (0.04) | 3,706 (1.68) |
|  | Chromosomes | 218,566 (98.84) | 96 (0.04) | 2,464 (1.11) |
|  | Polished chromosomes | 218,573 (98.85) | 99 (0.04) | 2,454 (1.11) |
|  | Final assembly | 219,013 (99.04) | 102 (0.05) | 2,011 (0.91) |
| exons | Contigs | 241,339 (98.10) | 489 (0.2) | 4,186 (1.70) |
|  | Chromosomes | 242,248 (98.47) | 509 (0.21) | 3,257 (1.32) |
|  | Polished chromosomes | 242,264 (98.48) | 512 (0.21) | 3,238 (1.32) |
|  | Final assembly | 242,971 (98.76) | 525 (0.21) | 2,518 (1.02) |
|  |  |  |  |  |
| Number of BUSCOs identified by polished assemblies | | |  |  |
| **Eukaryotic** (Total 303 searched) | |  |  |  |
| Polished assembly | #Complete (%) | #Fragmented (%) | #Missing (%) |  |
| MaSuRCA_hybrid | 261 (86.1) | 16 (5.3) | 26 (8.6) |  |
| FLYE_cor_ont | 263 (86.8) | 17 (5.6) | 23 (7.6) |  |
| FLYE_raw_ont | 267 (88.1) | 14 (4.6) | 22 (7.3) |  |
| WTDBG2_cor_ont | 261 (86.1) | 14 (4.6) | 28 (9.2) |  |
| WTDBG2_raw_ont | 259 (85.5) | 13 (4.3) | 31 (10.2) |  |
| MaSuRCA | 257 (84.8) | 22 (7.3) | 24 (7.9) |  |
| ALLPATHS | 241 (79.5) | 32 (10.6) | 30 (9.9) |  |
|  |  |  |  |  |
| **Vertebral** (Total 2,586 searched) | |  |  |  |
| Polished assembly | #Complete (%) | #Fragmented (%) | #Missing (%) |  |
| MaSuRCA_hybrid | 2,441 (94.4) | 71 (2.7) | 74 (2.9) |  |
| FLYE_cor_ont | 2,385 (92.2) | 133 (5.1) | 68 (2.6) |  |
| FLYE_raw_ont | 2,388 (92.3) | 118 (4.6) | 80 (3.1) |  |
| WTDBG2_cor_ont | 2,374 (91.8) | 114 (4.4) | 98 (3.8) |  |
| WTDBG2_raw_ont | 2,359 (91.2) | 114 (4.4) | 113 (4.4) |  |
| MaSuRCA | 2,392 (92.5) | 124 (4.8) | 70 (2.7) |  |
| ALLPATHS | 2,043 (79) | 383 (14.8) | 160 (6.2) |  |
|  |  |  |  |  |
| **Mammalian** (Total 4,104 searched) | |  |  |  |
| Polished assembly | #Complete (%) | #Fragmented (%) | #Missing (%) |  |
| MaSuRCA_hybrid | 3,880 (94.5) | 108 (2.6) | 116 (2.8) |  |
| FLYE_cor_ont | 3,801 (92.6) | 177 (4.3) | 126 (3.1) |  |
| FLYE_raw_ont | 3,793 (92.4) | 178 (4.3) | 133 (3.2) |  |
| WTDBG2_cor_ont | 3,785 (92.2) | 159 (3.9) | 160 (3.9) |  |
| WTDBG2_raw_ont | 3,783 (92.2) | 153 (3.7) | 168 (4.1) |  |
| MaSuRCA | 3,827 (93.3) | 151 (3.7) | 126 (3.1) |  |
| ALLPATHS | 3,273 (79.8) | 563 (13.7) | 268 (6.5) |  |

**Table S4. Results of fossil gene analysis for SIV *gag* and *pol* genes**

| **SIV *gag* gene EVEs (presented as number of amino acids, as compared to 658 amino acids encoded by *gag* genes downloaded from NCBI).** | | | |
| --- | --- | --- | --- |
| Chr | *M. fascicularis* | *M. m. mulatta* (Mmul_10 | *M. cyclopis* |
| 1 | Chr1:102,422,294-102,423,802 482/503 (96%)  Chr1:80,440,238-80,439,027 379/404 (94%) | Chr1:97,577,684-97,575,711 621/658 (94%)  Chr1:193,134,940-193,136,814 588/625 (94%)  Chr1: 25,811,260-25,809,365 587/632 (93%) | chr1:25,706,507-25,704,537 620/658 (94%) |
| 2 | Chr2:110,835,013-110,836,986 614/658 (93%)  Chr2:114,844,438-114,846,411 629/658 (96%)  Chr2:26,797,688-26,799,661 630/658 (96%)  Chr2:56,283,508-56,281,535 593/658 (90%)  Chr2:182,430,042-182,432,015 593/658 (90%)  Chr2:127,233,506-127,234,873 429/456 (94%) | Chr2:150,599,840-150,597,870 631/658 (96%)  Chr2:80,693,882-80,691,909 625/658 (95%)  Chr2:84,711,903-84,709,930 618/658 (94%)  Chr2:11,675,098-11,673,125 597/658 (91%)  Chr2:95,742,108-95,740,225 576/628 (92%)  Chr2:139,788,902-139,790,566 500/555 (90%) | Chr2:169,214,495-169,212,522 627/658 (95%) |
| 3 | Chr3:118,656,780-118,658,867 628/696 (90%)  Chr3:10,509,471-10,507,498 622/658 (95%)  Chr3:126,527,874-126,529,847 605/658 (92%)  Chr3:56,194,259-56,192,643 508/539 (94%) | Chr3:59,202,092-59,200,119 624/658 (95%)  Chr3:70,705,519-70,703,606 611/638 (96%)  Chr3:66,527,138-66,525,276 580/621 (93%)  Chr3:174,830,354-174,831,979 501/542 (92%) | Chr3:66,280,552-66,278,579 627/658 (95%) |
| 4 | Chr4:29,555,301-29,557,274 619/658 (94%)  Chr4:164,416,311-164,418,140 575/610 (94%) | Low Percentage and sequence Coverage. < 50% | Chr4:55,212,557-55,214,530 629/658 (96%) |
| 5 | Chr5:81,328,277-81,330,250 622/658 (95%)  Chr5:180,932,280-180,930,307 626/658 (95%)  Chr5:63,227,931-63,225,958 624/658 (95%)  Chr5:11,691,518-11,693,491 620/658 (94%)  Chr5:3,681,068-3,683,038 581/657 (88%)  Chr5:9,194,871-9,193,042 548/618 (89%) | Chr5:63,333,444-63,331,471 627/658 (95%)  Chr5:182,234,689-182,232,716 625/658 (95%)  Chr5:178,083,564-178,081,591 619/658 (94%)  Chr5: 3,632,732-3,634,705 584/658 (89%) | Chr5:63,281,780-63,279,807 628/658 (95%) |
| 6 | Chr6:40,048,991-40,050,964 632/658 (96%)  Chr6:60,576,198-60,577,964 551/589 (94%) | Chr6:62,009,437-62,011,407 600/658 (91%)  Chr6:62,031,668-62,033,434 556/589 (94%) | Chr6:39,980,943-39,982,916 627/658 (95%) |
| 7 | Chr7:148,148,688-148,146,715 622/658 (95%)  Chr7:76,369,184-76,367,211 626/658 (95%)  Chr7:166,523,993-166,525,966 610/658 (93%) | Chr7:63,503,349-63,501,376 629/658 (96%) | Chr7:63,933,368-63,931,395 627/658 (95%) |
| 8 | Chr8:38,882,112-38,884,085 622/658 (95%) | Chr8: 3,679,053- 3,677,152 603/634 (95%) | Chr8:39,420,098- 39,422,071 625/658 (95%) |
| 9 | Chr9:31,377,271-31,379,244 630/658 (96%)  Chr9:59,725,987-59,724,134 563/618 (91%) | Chr9:50,640,244-50,638,271 632/658 (96%)  Chr9:44,278,099-44,276,126 626/658 (95%)  Chr9:64,405,964-64,403,991 602/658 (91%)  Chr9:32,598,857-32,600,731 577/625 (92%) | Chr9:42,295,243-42,293,270 631/658 (96%) |
| 10 | Chr10:93,909,396-93,911,366 628/658 (95%)  Chr10:93,884,469-93,886,442 629/658 (96%)  Chr10:90,634,708-90,636,681 623/658 (95%)  Chr10:79,165,368-79,167,341 617/658 (94%)  Chr10:90,760,972-90,759,464 449/503 (89%) | Chr10:33,757,533-33,759,503 624/658 (95%)  Chr10: 33,732,888-33,734,858 551/658 (84%) | Chr10:33,108,433-33,110,406 628/658 (95%) |
| 11 | Chr11:90,990,981-90,989,008 626/658 (95%)  Chr11:99,064,187-99,066,160 628/658 (95%)  Chr11:128,671,180-128,669,207 620/658 (94%)  Chr11:35,550,103-35,548,232 584/624 (94%)  Chr11:28,536,366-28,535,032 387/454 (85%) | Chr11: 95,858,844-95,857,015 576/610 (94%) | Chr11:105,699,965-105,701,938; 627/658 (95%) |
| 12 | Chr12:27,143,964-27,141,991 626/658 (95%)  Chr12:122,688,408-122,689,784 432/459 (94%) | Chr12: 8,118,213-8,116,705 476/503 (95%) | Chr12:124,932,460-124,933,836; 429/459 (93%)  Chr12:29,597,754-29,596,612 360/381 (94%)  Chr12:78,435,915-78,435,508 125/136 (92%) |
| 13 | Chr13:87,190,711-87,192,669 624/658 (95%) | Chr13: 21,584,795- 21,582,900 589/632 (93%)  Chr13: 21,409,340- 21,407,448 587/632 (93%) | Chr13:22,247,155-22,245,182 622/658 (95%) |
| 14 | Chr14:37,305,820-37,307,793 633/658 (96%) | Chr14:89,624,457-89,626,430 632/658 (96%) | Chr14:89,806,047-89,804,074 633/658 (96%) |
| 15 | Chr15:82,158,749-82,156,776 633/658 (96%)  Chr15:19,769,208-19,767,241 630/658 (96%)  Chr15:93,445,087-93,443,414 423/559 (76%) | Chr15:31,236,243-31,238,216 633/658 (96%)  Chr15:18,507,813-18,509,684 585/624 (94%) | Chr15:29,324,832-29,326,805 633/658 (96%) |
| 16 | Chr16:55,088,085-55,086,577 476/503 (95%) | Chr16:32,523,278-32,525,251 624/658 (95%), | Chr16:32,402,051-32,404,024 623/658 (95%) |
| 17 | Chr17:44,952,758-44,950,785 626/658 (95%)  Chr17:43,753,513-43,754,934 411/474 (87%) | Chr17: 27,670,923-27,672,896 630/658 (96%) | Chr17:27,622,238-27,624,211 631/658 (96%) |
| 18 | Chr18:8,733,991-8,732,891 313/368 (85%) | Chr18: 8,889,820- 8,887,847 617/658 (94%)  Chr18: 47,195,513- 47,197,117 508/535 (95%) | Chr18:8,871,881-8,869,908 626/658 (95%) |
| 19 | Chr19:20,987,814-20,985,841 624/658 (95%)  Chr19:40,338,532-40,336,850 505/561 (90%) | Chr19:25,554,197-25,556,170 619/658 (94%)  Chr19:36,701,953-36,703,923 458/671 (68%) | Chr18:26,004,792-26,006,765 618/658 (94%) |
| 20 | Chr20:40,304,994-40,306,499 477/503 (95%) | Chr20: 40,450,799- 40,452,352 495/519 (95%) | Chr20:40,265,195-40,266,700 477/503 (95%)  Chr20:40,264,729-40,265,205 152/159 (96%) |
| X | ChrX: 50,949,416-50,947,443 628/658 (95%)  ChrX: 61,764,397-61,766,370 623/658 (95%)  ChrX: 57,579,780-57,578,272 470/503 (93%)  ChrX: 148,781,307-148,782,815 470/503 (93%) | ChrX: 51,136,296-51,134,323 626/658 (95%)  chrX:150,910,924-150,912,897  625/658 (95%)  ChrX: 58,485,958-58,483,985 618/658 (94%) | ChrX: 51,060,647-51,058,674 626/658 (95%) |
| **Sum** | **56** | **44** | **24** |
| **SIV *pol* gene EVEs (presented as number of amino acids, as compared to 574 amino acids encoded by the *pol* gene downloaded from NCBI).** | | | |
|  | *M. fascicularis* | *M. m. mulatta* (Mml_10) | *M. cyclopis* |
| 1 | Chr1:102,424,545-102,426,251 541/569 (95%)  Chr1:80,438,284-80,436,578 539/569 (95%)  Chr1:174,647,248-174,645,788 460/487 (94%)  Chr1:103,100,611-103,102,071 458/487 (94%) | Chr1:97,574,968-97,573,265 494/586 (84%)  Chr1:193,137,768-193,139,264 471/499 (94%)  Chr1:104,412,745-104,414,205 458/487 (94%)  Chr1:25,808,174-25,806,840 400/446 (90%) | Chr1:82,401,887-82,400,181 540/569 (95%) |
| 2 | Chr2:114,847,154-114,848,860 537/569 (94%)  Chr2:110,837,727-110,839,433 536/569 (94%)  Chr2:182,432,759-182,434,465 534/569 (94%)  Chr2:127,234,865-127,236,490 512/542 (94%)  Chr2:56,280,804-56,279,104 510/567 (90%)  Chr2:26,800,624-26,802,102 468/493 (95%) | Chr2:80,691,166-80,689,460 538/569 (95%)  Chr2:31,091,835-31,093,541 537/569 (94%)  Chr2:150,597,127-150,595,421 535/569 (94%)  Chr2:11,672,344-11,670,677 521/556 (94%)  Chr2:139,791,601-139,793,301 511/567 (90%)  Chr2:84,709,187-84,707,466 511/575 (89%)  Chr2:169,343,996-169,342,518 469/493 (95%) | chr2:31,135,258-31,136,964 540/569 (95%) |
| 3 | Chr3:10,506,756-10,505,050 540/569 (95%)  Chr3:8,783,063-8,784,769 538/569 (95%)  Chr3:118,659,607-118,661,313 534/569 (94%)  Chr3:84,946,828-84,948,288 458/487 (94%)  Chr3:126,530,762-126,532,294 456/511 (89%) | Chr3:108,207,883-108,209,589 541/569 (95%)  Chr3:174,832,799-174,834,505 537/569 (94%)  Chr3:70,702,805-70,701,099 535/569 (94%)  Chr3:66,524,424-66,522,721 536/569 (94%) | Chr3:174,473,283-174,474,989 541/569 (95%) |
| 4 | Chr4:30,983,130-30,981,424 539/569 (95%)  Chr4:29,558,017-29,559,723 537/569 (94%)  Chr4:8,325,433-8,326,893 456/487 (94%) | Low percentage and sequence coverage. < 50% | Chr4:139,318,427-139,316,721 533/569 (94%)  Chr4:26,104,243-26,102,768 427/493 (87%) |
| 5 | Chr5:81,330,993-81,332,699 541/569 (95%)  Chr5:53,677,068-53,675,362 541/569 (95%)  Chr5:11,694,234-11,695,940 535/569 (94%)  Chr5:180,929,564-180,927,855 536/570 (94%)  Chr5:63,225,214-63,223,508 533/569 (94%)  Chr5:3,683,784-3,685,490 502/569 (88%)  Chr5:132,417,904-132,419,364 460/487 (94%)  Chr5:3,481,854-3,480,574 459/487 (94%) | Chr5:53,712,435-53,710,729 539/569 (95%)  Chr5:178,080,849-178,079,143 541/569 (95%)  Chr5:63,330,728-63,329,022 536/569 (94%)  Chr5:182,231,973-182,230,264 535/570 (94%)  Chr5:3,635,448-3,637,154 503/569 (88% | Chr5:53,677,774-53,676,068 534/569 (94%) |
| 6 | Chr6:40,051,707-40,053,413 542/569 (95%)  Chr6:60,578,707-60,580,413 534/569 (94%) | Chr6:135,181,723**:**135,180,017 538/569 (95%)  Chr6:62,034,177-62,035,883 532/569 (93%)  Chr6:62,012,243-62,013,865 484/542 (89%)  Chr6:83,353,527-83,354,987 460/487 (94%) | Chr6:134,814,290-134,812,584; 538/569 (95%) |
| 7 | Chr7:76,366,468-76,364,762 542/569 (95%)  Chr7:166,526,709-166,528,415 539/569 (95%)  Chr7:148,145,972-148,144,266 535/569 (94%)  Chr7:79,594,250-79,592,544 528/569 (93%) | Chr7:63,500,633-63,498,927 545/569 (96%)  Chr7:78,937,153-78,938,613 459/487 (94%)  Crh7:99,037,294-99,038,616 327/446 (73%) | Chr7:63,930,652-63,928,946 541/569 (95%) |
| 8 | Chr8:44,616,378-44,618,084 540/569 (95%)  Chr8:38,884,828-38,886,534 536/569 (94%)  Chr8:40,060,765-40,062,225 461/487 (95%) | Chr8:3,676,333-3,674,627 538/569 (95%)  Chr8:47,139,090-47,140,796 536/569 (94%) | Chr8:46,185,017-46,186,723 539/569 (95%) |
| 9 | Chr9:31,379,987-31,381,693 537/569 (94%) | Chr9:50,637,528-50,635,822 540/569 (95%)  Chr9:32,601,474-32,603,180 538/569 (95%)  Chr9:44,275,062-44,273,356 537/569 (94%)  Chr9:37,109,684-37,108,254 449/477 (94%)  Chr9:133,537,883-133,539,352 436/492 (89%) | Chr9:42,292,527-42,290,821 542/569 (95%) |
| 10 | Chr10:93,887,185-93,888,891 539/569 (95%)  Chr10:93,912,422-93,914,128 540/569 (95%)  Chr10:79,168,084-79,169,790 535/569 (94%)  Chr10:90,637,424-90,639,130 533/569 (94%)  Chr10:90,758,721-90,757,015 528/569 (93%) | Chr10:33,735,601-33,737,307 537/569 (94%)  Chr10:36,335,817-36,337,079 388/421 (92%) | Chr10:33,111,149-33,112,855; 538/569 (95%) |
| 11 | Chr11:99,066,903-99,068,609 542/569 (95%)  Chr11:90,988,265-90,986,559 538/569 (95%)  Chr11:35,547,486-35,545,783 534/568 (94%)  Chr11:28,533,651-28,532,311 408/447 (91%) | Chr11:101,903,303-101,905,009 540/569 (95%)  Chr11:95,856,130-95,854,424 537/569 (94%)  Chr11:29,821,883-29,820,177 532/569 (93%) | Chr11:105,702,681-105,704,387 542/569 (95%) |
| 12 | Chr12:5,369,597-5,371,303 539/569 (95%)  Chr12:122,690,527-122,692,233 538/569 (95%)  Chr12:27,141,248-27,139,542 535/569 (94%)  Chr12:127,707,831-127,706,371 459/487 (94%)  Chr12:76,105,582-76,104,092 459/498 (92%) | Chr12:29,488,239-29,486,713 480/509 (94%)  Chr12:90,217,947-90,219,407 458/487 (94%)  Chr12:78,396,544-78,395,114 441/477 (92%)  Chr12:8,115,492-8,114,257 376/412 (91%) | Chr12:29,595,406-29,593,700 538/569 (95%) |
| 13 | Chr13:87,193,412-87,195,118 536/569 (94%)  Chr13:7,360,060-7,358,633 416/477 (87%) | Chr13:33,811,575-33,810,115 461/487 (95%)  Chr13:21,581,709-21,580,375 410/446 (92%)  Chr13:98,973,136-98,974,446 356/440 (81%) | Chr13:12,582,519-12,580,813; 536/569 (94%) |
| 14 | Chr14:37,308,536-37,310,242 538/569 (95%) | Low percentage and sequence coverage. < 50% | Chr14:89,803,331-89,801,625; 541/569 (95%) |
| 15 | Chr15:82,156,033-82,154,327 540/569 (95%)  Chr15:19,766,501-19,764,753 530/583 (91%)  Chr15:93,442,474-93,440,765 528/570 (93%)  Chr15:81,087,915-81,089,375 460/487 (94%)  Chr15:71,757,137-71,755,677 456/487 (94%) | Chr15:31,238,959-31,240,665 541/569 (95%)  Chr15:45,348,485-45,350,191 534/569 (94%)  Chr15:18,510,427-18,512,133  535/569 (94%) | Chr15:29,327,548-29,329,254; 539/569 (95%) |
| 16 | Chr16:55,085,834-55,084,128 538/569 (95%)  Chr16:57,246,628-57,244,922 535/569 (94%)  Chr16:69,548,974-69,547,379 505/532 (95%) | Chr16:32,525,994-32,527,670 530/559 (95%)  Chr16:20,236,944-20,238,404 460/487 (94%) | Chr16:7,778,294-7,780,000 540/569 (95%) |
| 17 | Chr17:43,755,677-43,757,383 539/569 (95%)  Chr17:61,950,282-61,948,822 455/487 (93%)  Chr17:44,949,572-44,948,334 361/413 (87%) | Chr17:35,266,892-35,268,352 458/487 (94%)  Chr17:63,837,681-63,836,221 456/487 (94%)  Chr17:27,673,639-27,674,835 307/404 (76%) | Chr17:43,599,272-43,600,978 538/569 (95%) |
| 18 | Low percentage and sequence coverage. < 50% | Chr18:47,198,225-47,199,931 537/569 (94%)  Chr18:8,887,104-8,885,401 514/569 (90%) | Chr18:8,869,165-8,867,459 540/569 (95%) |
| 19 | Chr19:20,985,098-20,983,392 538/569 (95%)  Chr19:40,335,814-40,334,108 528/569 (93%)  Chr19:35,113,560-35,115,260 519/567 (92%)  Chr19:11,923,085-11,924,545 459/487 (94%)  Chr19:22,889,822-22,891,525 465/578 (80%) | Chr19:36,704,667-36,706,373 529/569 (93%)  Chr19:22,140,081-22,141,595 464/505 (92%)  Chr19:58,020,772-58,019,567 377/402 (94%) | Chr19:58,665,572-58,667,278 537/569 (94%) |
| 20 | Chr20:31,411,685-31,413,391 535/569 (94%)  Chr20:40,307,242-40,308,948 536/569 (94%) | Chr20:40,453,510-40,454,709 377/400 (94%) | Chr20:40,267,443-40,269,149 541/569 (95%) |
| X | ChrX: 50,946,700-50,944,994 539/569 (95%)  ChrX: 57,577,529-57,575,823 539/569 (95%)  ChrX: 148,783,559-148,785,265 539/569 (95%)  ChrX: 121,221,346-121,222,806 460/487 (94%)  ChrX: 76,953,048-76,951,588 456/487 (94%) | ChrX: 51,133,585-51,131,879 540/569 (95%)  ChrX: 150,913,640-150,915,346 538/569 (95%)  ChrX: 58,483,242-58,481,536 539/569 (95%)  ChrX: 153,116,791-153,115,085 535/569 (94%)  ChrX: 118,840,270-118,841,733 463/488 (95%) | ChrX: 51,057,931-51,056,225; 541/569 (95%)  ChrX: 78,207,014-78,205,545; 437/492 (89%) |
| **Sum** | **76** | **64** | **23** |
